# The moderating role of diet and physical activity in insulin resistance and immunometabolic depression

**DOI:** 10.1038/s41598-025-32454-4

**Published:** 2025-12-23

**Authors:** Judith R. Gruber, Carmen Schiweck, Alea Ruf, Elena D. Süß, Philipp Japtok, Niklas Schouler, Sharmili Edwin Thanarajah, Andreas Reif, Silke Matura

**Affiliations:** 1 Department of Psychiatry, Psychosomatic Medicine and Psychotherapy, University Hospital, Goethe University Frankfurt, Heinrich-Hoffmann-Straße 10, 60528 Frankfurt am Main, Germany; 2 Department of Clinical and Health Psychology, University of Vienna, Vienna, Austria; 3 Mental mHealth Lab, Institute of Sports and Sports Science, Karlsruhe Institute of Technology (KIT), Karlsruhe, Germany; 4 Department of General Internal Medicine and Psychosomatics, University Hospital Heidelberg, Heidelberg, Germany; 5 Fraunhofer Institute for Translational Medicine and Pharmacology ITMP, Theodor-Stern-Kai 7, 60596 Frankfurt am Main, Germany; 6 Max Planck Institute for Metabolism Research, Cologne, Germany

**Keywords:** Inflammation, Diet, Mental health, Physical activity, Insulin resistance, Immunology, Psychology, Endocrinology, Risk factors

## Abstract

Insulin resistance (IR) and atypical depression share an immunometabolic pathway and present major health risks. Although IR has been associated with atypical depression, its link to immunometabolic depression (IMD) symptoms is less understood. Lifestyle factors like physical activity (PA) and diet may mitigate these conditions through anti-inflammatory and antidiabetic effects. This study examines how lifestyle factors moderate the IR-IMD relationship. We conducted a cross-sectional analysis using baseline data from the *m*PRIME study (n = 124). IR was measured via HOMA-IR, and IMD symptoms (hypersomnia, hyperphagia, loss of energy, tiredness) were assessed using a composite score derived from four Beck Depression Inventory-II items that have been previously linked to immunometabolic alterations. PA was tracked using accelerometers; diet via the empirical dietary inflammatory pattern (EDIP). Multiple linear regression and moderation analyses were applied. Ninety-four individuals (M(age) = 49.43, SD = 13.93; 48% IR) were analyzed. IR was significantly associated with the IMD-score (*β* = 0.817, *p* = 0.001). PA showed no direct or moderating effect (*p* > 0.05). A proinflammatory diet moderated the IR-IMD link in men (*β* = 0.718, *p* < 0.001), but not women (*p* > 0.05). Findings indicate an IR-IMD association, suggesting symptom monitoring in IR patients may aid early IMD detection and prevention.

*Trial registration* Registered at https://www.drks.de/search/de/trial/DRKS00022774 Identifier no. DRKS00022774 (Registration date: 2021-03-08).

**Supplementary Information:**

The online version contains supplementary material available at 10.1038/s41598-025-32454-4.

## Introduction

Modifiable risk factors such as physical activity (PA) and diet are crucial for the prevention and treatment of type 2 diabetes and depressive symptoms^1–3^. Type 2 diabetes, a leading cause of disability-adjusted life years, affects approximately 529 million individuals globally, with men being 1.14 times more likely to be affected^4^. A well-established bidirectional relationship exists between type 2 diabetes and depression^5,6^, with depressive symptoms often emerging in stages of insulin resistance (IR)^7–9^. Low-grade inflammation is a key mechanism linking these conditions^9–12^. Hence, lifestyle factors such as diet and PA, which have anti-inflammatory potential, appear to play a role in the prevention of these comorbidities^13–16^.

A meta-analysis^7^, including 25 case-control and cross-sectional studies, revealed a significant association between depressive symptoms and IR. Additionally, genetic overlap between insulin-related diseases and major depressive disorder (MDD) has been identified^17^. Recent findings indicate that this relationship is particularly pronounced for somatic symptoms of depression^12,18–20^ and even more pronounced for symptoms of atypical depression, such as hypersomnia (increased sleep) and hyperphagia (increased appetite)^9,11,21^. For example, hyperphagia and hypersomnia were found to be positively associated with IR and BMI in primary care patients with depression but without diabetes^12^. Conversely, poor appetite was linked to lower IR, and disturbed sleep was not associated with IR^12^. Consistently, a meta-analysis^9^ revealed elevated insulin levels in individuals with acute depression, specifically within the atypical subtype, supporting the hypothesis of a metabolic subgroup. Further delineation of a subgroup of patients with depression, who display abnormalities in insulin signaling and/or IR, might pave the way for precision medicine approaches^22^. This is highly relevant, as depression as a whole is highly heterogeneous which also explains the lack of progress regarding new depression treatments^23^.

Several studies have indicated that elevated levels of inflammation, e.g., increased levels of C-reactive protein (CRP), and metabolic markers are specific for certain somatic symptoms of depression^12,24–27^. Research by Milaneschi et al.^28^ suggested that immunometabolic dysregulations are not associated with depression or the atypical subtype per se, but are specifically linked to symptoms of atypical depression associated with altered energy intake. The concept is referred to as immunometabolic depression (IMD) and is characterized by behavioral symptoms such as increased appetite, weight gain, hypersomnia, fatigue and leaden paralysis^28^. Although IMD shares several clinical features with atypical depression it represents a more biologically defined construct. While atypical depression is categorized based on symptom presentation, IMD specifically refers to a depression subtype characterized by concurrent immunometabolic dysregulations, including low-grade inflammation, altered insulin signaling and metabolic disturbances^28,29^. Individuals with IMD are more likely to be female, have an earlier age at onset and exhibit a higher BMI^29^. Studies have confirmed the concept of IMD by showing that inflammation (CRP and IL-6 concentrations) is prevalent in individuals with MDD who report low energy levels together with increased appetite and sleep^30,31^. Furthermore, Lamers et al.^32^ identified increased appetite as the only depressive symptom associated with increased inflammation and components of the metabolic syndrome, such as increased waist circumference, triglycerides, blood pressure, fasting plasma glucose and decreased HDL-cholesterol, potentially making it a core symptom of IMD^32^. With respect to IR, a study revealed higher levels of HOMA-IR and immunometabolic dysregulations in an MDD group with increased appetite than in an MDD group with decreased appetite or healthy controls^33^.

Reduced PA (partly due to hypersomnia) and increased food intake (due to hyperphagia) likely contribute to increases in adipose tissue and thereby, increase both inflammation and IR. Under normal conditions, immune cells regulate and ensure homeostasis in adipose tissue. Increased adipose tissue and an excess of free fatty acids and hyperglycemia can lead to increased immune cell attraction and a proinflammatory environment through the activation of distinct pathways (i.e., Jun Kinase and IKK-β pathways)^34^. The proinflammatory environment, in turn, shifts the phosphorylation of the insulin receptor substrate (IRS) from tyrosine to serine phosphorylation, which ultimately reduces the action of insulin. This can then further increase both inflammation and IR, potentially leading to aggravation of the previously mentioned depressive symptoms. Exploring relationships among these factors is therefore crucial for devising integrative treatment approaches for individuals with IR who might be at risk for IMD. Furthermore, individuals with depressive symptoms including more IMD features showed less improvement during antidepressant treatment, likely due to the influence of elevated CRP levels^35^. These findings indicate that these individuals may benefit from lifestyle modifications that show anti-inflammatory potential.

The World Health Organization recently reported that one-third of the world’s adult population does not meet the recommendations for PA (150 min of moderate intensity or 75 min of vigorous intensity per week)^36,37^, with inactivity being more prevalent in females. PA is an integral component of diabetes treatment guidelines because it improves insulin sensitivity and reduces abdominal fat^1^. Furthermore, physical exercise reduces oxidative stress, inflammation, cortisol levels and the risk of metabolic syndrome^13,38,39^. An interventional study comparing antidepressant therapy with physical exercise reported comparable effectiveness in reducing depression, with further improvements in metabolic markers in the exercise group^40^. In line with this, a meta-analysis of 15 prospective studies, showed that even PA below the recommended levels has positive effects on depression^2^. With regard to atypical depression, studies indicate an association with physical inactivity, poor diet quality and increased risk of overweight^41–43^, emphasizing the potential of lifestyle interventions in managing this condition.

Similarly, proinflammatory diets have been linked to depressive symptoms^15,16,44^. Additionally, research has shown that a greater intake of proinflammatory foods is associated with increased IR^45,46^. Two widely used dietary indices to capture proinflammatory diets are the empirical dietary inflammatory pattern (EDIP) and the dietary inflammatory index (DII). Both indices have been shown to significantly predict concentrations of CRP and IL-6^47^. The EDIP has the advantage that it can be easily replicated in a standardized way and that its dietary pattern can be easily translated into recommendations^48^. While the DII is commonly used to capture the inflammatory potential of the diet in the context of depression, alternative scores have shown similar results^49,50^.

Although research on the relationship between IR and atypical depression has gained much attention, research linking IR to IMD symptoms remains limited. Although both IR and depression are independently associated with a pro-inflammatory lifestyle, few studies have examined the interaction of these factors.

First results of the *m*PRIME study revealed that insulin-resistant individuals demonstrated lower adherence to healthy lifestyle behaviors than insulin-sensitive individuals, which was attributed primarily to lower PA levels in the IR group^51^. In addition, in the IR group, inflammation, measured by the CRP level, was associated with lower levels of PA, indicating the potential of PA for prevention^51^. The objectives of this study are to explore (1) the association between IR and IMD symptoms and (2) the influence of proinflammatory diets and PA on IMD symptoms and (3) moderating effects of lifestyle factors on the relationship between IR and IMD symptoms. In consideration of the significant sex differences reported in previous studies, we also aimed to investigate sex-specific associations. To address these research questions, we used data from the *m*PRIME study. By exploring the interaction of lifestyle factors and IR in the context of IMD symptoms, we aim to provide initial indicators for the preventative potential of lifestyle modifications in individuals with comorbid IR and IMD.

## Results

### Descriptive statistics

In total, 94 individuals with a mean age of 49.43 ± 13.93 years were included in the analyses. The sample comprised 57% (*n* = 54) women and 43% (*n* = 40) men. In the total sample, the average BDI-II total score was 5.35 ± 6.48, indicating low depressive symptoms. Symptoms of moderate depression (BDI-II total score ≥ 14) were present in 13% (*n* = 12) of the participants. No sex differences emerged for sociodemographic, depressive or metabolic characteristics other than for education (*p* = 0.050) and waist circumference (*p* = 0.047). On average, men had more years of education and a greater waist circumference than women did. All descriptive statistics for the total sample and for each sex are shown in Table 1.

**Table 1:** Baseline sociodemographic characteristics.

	All (N = 94)	Men (n = 40)	Women (n = 54)	*p- v*alue^a,b^
Age, years	49.43 ± 13.93	49.00 ± 15.25	49.74 ± 13.0	0.754
Education, years	15.61 ± 2.94	16.20 ± 3.07	15.17 ± 2.78	0.050*
BMI, kg/m^2^	28.06 ± 5.88	27.62 ± 4.76	28.39 ± 6.62	0.945
BMI ≤ 25	35 (37)	13 (32)	22 (41)	0.238
BMI 25 to 29.9	31 (33)	17 (42)	14 (26)	
BMI ≥ 30	28 (30)	10 (25)	18 (33)	
Waist circumference, cm	96.07 ± 16.01	99.70 ± 13.50	93.39 ± 17.27	0.047*
CRP, mg/dl	0.09 [0.05–0.21]	0.09 [0.04–0.16]	0.10 [0.05–0.27]	0.223
IL-6^a^, pg/mL	2.32 [1.41–3.50]	2.72 [1.55–3.34]	2.09 [1.40–3.67]	0.503
HbA_1c_, %	5.49 ± 0.40	5.51 ± 0.41	5.48 ± 0.39	0.786
Untreated diabetes, *n*	3 (3)	2 (5)	1 (2)	0.791
HOMA-IR	2.53 ± 1.84	2.40 ± 1.61	2.61 ± 2.01	0.810
Smoking, *n*				
Non-smoker	82 (87)	33 (83)	49 (91)	0.384
Current smoker	12 (13)	7 (17)	5 (9)	
Moderate depression, *n*^c^	12 (13)	3 (8)	9 (17)	0.225
Antidepressants, *n*	4 (4)	1 (3)	3 (6)	0.834
BDI-II total score	5.35 ± 6.48	4.32 ± 6.35	6.11 ± 6.52	0.141

Data are presented as the means $\pm$ SDs, medians [Interquartile ranges], or *n* (%)*.* BDI-II, Beck Depression Inventory-II; BMI, body mass index; CRP, C-reactive protein; HOMA-IR, homeostasis model assessment of insulin resistance; SD, standard deviation; ^a^Pearscon chi-square test for categorical variables. ^b^Mann-Whitney U test for continuous variables. ^c^BDI-II total score ≥ 14. *p <.05.

^a^Data available for 83 individuals (men: *n* = 33, women: *n* = 50).

### Descriptive lifestyle and IMD statistics

Table 2 shows the descriptive statistics for the IMD-score components and lifestyle factors included as dependent and independent variables in the multiple linear regression models. At the descriptive level, women had slightly higher total and individual symptom scores than men did, but the differences were not statistically meaningful. The lifestyle factor EDIP ranged from −3.31 to 2.31 and MVPA ranged from 0.00 to 211.60 min/day in the total sample.

**Table 2:** Descriptive statistics of IMD components and lifestyle factors.

Variable	All			Men			Women			*p-*value^a^
	M	SD	Range	M	SD	Range	M	SD	Range	
IMD-score	1.20	1.32	0–5	0.92	1.19	0–4	1.41	1.39	0–5	0.073
Hypersomnia	0.22	0.47	0–2	0.17	0.45	0–2	0.26	0.48	0–2	0.300
Hyperphagia	0.12	0.44	0–3	0.07	0.35	0–2	0.15	0.49	0–3	0.308
Loss of energy	0.46	0.56	0–2	0.38	0.49	0–1	0.52	0.61	0–2	0.299
Tiredness	0.40	0.55	0–2	0.30	0.46	0–1	0.48	0.61	0–2	0.163
EDIP	−0.21	0.97	−3.31–2.31	−0.35	1.18	−3.31–2.31	−0.11	0.78	−2.80–2.03	0.614
MVPA, min/day	62.22	53.35	0.00–211.60	69.17	45.03	0.40–173.14	57.07	58.64	0.00–211.60	0.053

EDIP, empirical dietary inflammatory pattern; IMD-score, immunometabolic depression score; MPVA, moderate-to-vigorous physical activity. ^a^Mann-Whitney U test.

### Correlations between lifestyle factors, inflammatory markers and IMD

**Fig. 1:**
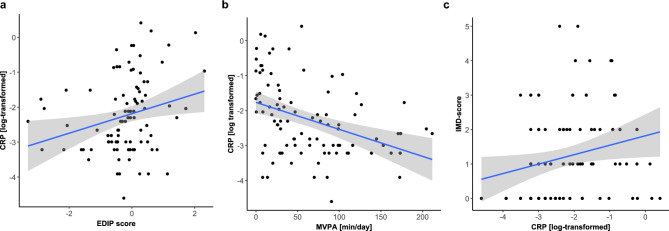
Relationships between CRP levels, lifestyle factors and IMD-score. Association between: (**a**) EDIP (empirical dietary inflammatory pattern) and log-transformed CRP levels, (**b**) MVPA (oderate-to-vigorous physical activity) and log-transformed CRP levels, (**c**) log-transformed CRP levels and IMD-score (immunometabolic depression score). All plots include a linear regression line with 95% confidence interval.

EDIP and MVPA were used as proxies for the inflammatory potential of lifestyle, therefore their correlations with CRP and IL-6 were investigated. CRP was positively correlated with a proinflammatory diet (*r*_*s*_ = 0.279, *p* = 0.006) and inversly correlated with PA (*r*_*s*_ = −0.435, *p* < 0.001). The IMD-score was also moderately correlated with the CRP level (*r*_*s*_ = 0.295, *p* = 0.004). These relationships are depicted in Fig. 1, which shows scatterplots for CRP and lifestyle factors (EDIP, MVPA) and for CRP and the IMD-score. For IL-6, a proinflammatory diet showed no correlation (*r*_*s*_ = 0.184, *p* = 0.096), whereas PA showed a moderate inverse relationship (*r*_*s*_ = −0.387, *p* < 0.001). The IMD-score also showed no correlation with IL-6 (*r*_*s*_ = 0.190, *p* = 0.085). An additional figure illustrating the relationships between IL-6 and lifestyle factors (EDIP, MVPA) and between IL-6 and the IMD-score is available in the Online Appendix [see Fig. S1]. The detailed results of the correlation matrix are also shown in the Online Appendix [see Table S1].

### Associations between IR, lifestyle and IMD

The effects of IR and lifestyle on IMD were tested, and the results of the multiple linear regression models are displayed in Table 3. Model 1 revealed that in the total sample, an increase in the HOMA-IR score by one unit was associated with an increase in the IMD-score of 0.82 points, *β* = 0.817, *p* = 0.001. Sex was also a significant predictor of IMD, with women showing greater IMD-scores than men, *β* = 0.808, *p* = 0.018. Neither EDIP nor MVPA were significant predictors of IMD, *p* = 0.548 and *p* = 0.410, respectively.

**Table 3:** Model estimates of multiple linear regression models.

Effect	All					Male					Female				
	Estimate	SE	95% CI		*P*-Value	Estimate	SE	95% CI		*P*-Value	Estimate	SE	95% CI		*P*-Value
			LL	UL				LL	UL				LL	UL	
*Model 1 – IMD-score*	R^2^ = 0.230 (*P* = 0.007)					R^2^ = 0.381 (*P* = 0.010)					R^2^ = 0.266 (*P* = 0.063)				
Intercept	1.670	0.945	−0.209	3.549	0.136	0.236	1.357	−2.525	2.996	0.863	3.466	1.269	0.909	6.022	0.009*
HOMA-IR	0.817	0.231	0.358	1.275	0.001*	0.816	0.242	0.325	1.307	0.002*	0.9780	0.373	0.229	1.731	0.012*
EDIP	0.088	0.177	−0.264	0.441	0.548	0.294	0.153	−0.017	0.606	0.063	−0.004	0.004	−0.012	0.004	0.342
MVPA	−0.003	0.003	−0.009	0.004	0.410	0.002	0.006	−0.009	0.013	0.710	−0.362	0.220	−0.805	0.082	0.108
Age	0.000	0.011	−0.021	0.021	0.999	0.008	0.011	−0.015	0.031	0.484	−0.024	0.023	−0.070	0.023	0.309
Smoking	0.393	0.390	−0.383	1.169	0.325	−0.064	0.467	−1.015	0.887	0.892	1.198	0.837	−0.487	2.883	0.159
BMI	−0.041	0.036	−0.113	0.031	0.225	−0.010	0.053	−0.117	0.098	0.858	−0.045	0.037	−0.119	0.029	0.223
Contraception	−0.576	1.084	−2.731	1.579	0.349						−0.692	1.164	−3.037	1.652	0.555
Menopause	−0.690	0.440	−1.566	0.186	0.110						−0.312	0.534	−1.387	0.763	0.562
Sex	0.808	0.352	0.109	1.507	0.018*										
*Model 2 – IMD-score*	R^2^ = 0.230 (*P* = 0.012)					R^2^ = 0.515 (*P* = 0.001)					R^2^ = 0.3126 (*P* = 0.038)				
Intercept	1.678	0.961	−0.234	3.589	0.085	1.513	1.084	−0.695	3.722	0.172	4.779	1.328	2.103	7.455	0.001*
HOMA-IR	0.816	0.238	0.345	1.289	0.001*	0.976	0.193	0.583	1.369	0.000*	0.901	0.366	0.164	1.639	0.018*
EDIP	0.100	0.217	−0.331	0.531	0.646	−0.142	0.137	−0.422	0.137	0.307	0.663	0.584	−0.514	1.841	0.262
MVPA	−0.003	0.003	−0.009	0.004	0.434	−0.003	0.005	−0.012	0.007	0.567	−0.007	0.004	−0.068	0.023	0.110
Age	−0.000	0.012	0.023	0.023	0.997	0.019	0.010	−0.003	0.040	0.084	−0.022	0.023	−0.068	0.023	0.331
Smoking	0.386	0.380	−0.369	1.142	0.312	0.267	0.420	−0.590	1.123	0.530	0.400	0.791	−1.195	1.995	0.616
BMI	−0.041	0.039	−0.118	0.036	0.293	−0.076	0.042	−0.161	0.010	0.081	−0.077	0.041	−0.159	0.005	0.065
HOMA-IR*EDIP	−0.013	0.334	−0.678	0.652	0.968	0.718	0.135	0.442	0.993	0.000*	−0.867	0.501	−1.876	0.142	0.091
Contraception	−0.577	1.058	−2.682	1.528	0.587						−0.860	1.091	−3.060	1.339	0.435
Menopause	−0.691	0.467	−1.620	0.237	0.142						−0.497	0.583	−1.671	0.678	0.400
Sex	0.805	0.348	0.112	1.498	0.020*										
*Model 3 – IMD-score*	R^2^ = 0.233 (*P* = 0.010)					R^2^ = 0.382 (*P* = 0.021)					R^2^ = 0.289 (*P* = 0.064)				
Intercept	1.820	0.978	−0.126	3.765	0.066	0.307	1.602	−2.956	3.570	0.849	4.020	1.360	1.278	6.761	0.005
HOMA-IR	0.993	0.403	0.192	1.794	0.016*	0.951	0.732	−0.539	2.442	0.203	1.344	0.535	0.267	2.422	0.016
EDIP	0.090	0.179	−0.265	0.445	0.616	0.297	0.161	−0.030	0.625	0.074	−0.362	0.214	−0.793	0.070	0.099
MVPA	−0.002	0.003	−0.008	0.005	0.554	0.003	0.008	−0.013	0.019	0.728	−0.003	0.004	−0.012	0.006	0.497
Age	−0.002	0.011	−0.023	0.019	0.873	0.008	0.012	−0.016	0.031	0.511	−0.032	0.023	−0.079	0.014	0.169
Smoking	0.374	0.379	−0.380	1.129	0.327	−0.063	0.485	−1.051	0.926	0.898	0.951	0.820	−0.701	2.604	0.252
BMI	−0.046	0.038	−0.122	0.030	0.232	−0.015	0.069	−0.155	0.126	0.835	−0.059	0.040	−0.139	0.021	0.144
HOMA-IR*MVPA	−0.002	0.004	−0.011	0.006	0.568	−0.001	0.010	−0.021	0.018	0.881	−0.007	0.006	−0.018	0.005	0.235
Contraception	−0.559	1.062	−2.672	1.554	0.600						−0.622	1.071	−2.781	1.537	0.564
Menopause	−0.661	0.436	−1.528	0.206	0.133						−0.143	0.532	−1.215	0.929	0.789
Sex	0.751	0.339	0.076	1.426	0.030*										

EDIP, Empirical dietary inflammatory pattern; HOMA-IR, homeostasis model assessment of insulin resistance; IMD-score, immunometabolic depression score; MPVA, moderate-to-vigorous-physical-activity. Sex (0 = male, 1 = female); smoking (0 = non-smoker, 1 = smoker), contraception (0 = no intake, 1 = intake) and menopause (0 = no, 1 = yes) *p < 0.05.

### Effect modification of lifestyle on IR and IMD

**Fig. 2:**
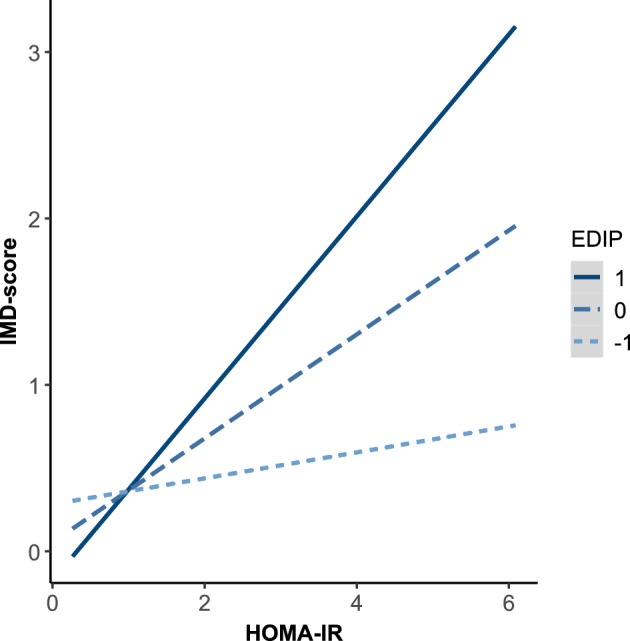
Interaction Plot between HOMA-IR and EDIP in men. The x-axis represents HOMA-IR, while the y-axis shows the IMD-score (immunometabolic depression score). The interaction is shown for different EDIP (empirical dietary inflammatory pattern) scores defined as −1 (low), 0 (neutral) and + 1 (high). The graph illustrates how variations in EDIP influence the relationship between HOMA-IR and IMD-score with separate regression lines for each score of EDIP.

Furthermore, the effect of lifestyle on the relationship between IR and IMD was explored in Models 2 and 3 (Table 3). In the total sample, there was no interaction between HOMA-IR and EDIP. However, in men, there was an interaction effect between HOMA-IR and EDIP (*β* = 0.718, *p* < 0.001). Thus, among men a more proinflammatory diet strengthened the influence of IR on IMD. The interaction is illustrated in Fig. 2, which shows the relationship between HOMA-IR and the IMD-score for different EDIP scores in men. In women, no interaction between HOMA-IR and EDIP was observed. Interaction plots for the total sample and the female sample can be found in the Appendix [see Figs. S2 and S3]. In addition, PA did not modify the effect of IR on IMD symptoms (Model 3) and no sex-specific effects were observed (*p* > 0.05).

## Discussion

Using objectively and longitudinally assessed PA, detailed dietary data, and an IMD-score we investigated the modifying effects of lifestyle on IR-associated depressive symptoms. We found an association between IR and IMD symptoms. Furthermore, CRP was correlated with IMD symptoms. However, neither a proinflammatory diet nor PA predicted IMD symptoms in the overall sample, and PA did not moderate the effect of IR on IMD symptoms. Interestingly, however, in males, IR was associated with IMD symptoms and a proinflammatory diet strengthened this association. In females, no such interaction occured. These results suggest that men may particularly benefit from an anti-inflammatory diet.

Our findings of an association between IR and IMD symptoms align with studies linking IR-related disorders and symptoms of atypical depression^9,11,21,52^. Using a literature-based method to combine four depressive symptoms (hypersomnia, hyperphagia, loss of energy and tiredness) associated with metabolic and inflammatory changes, we adopted a holistic approach rather than only looking at individual symptoms^12,53^. Fernandes et al.^9^ proposed the use of HOMA-IR and insulin levels as biomarkers for the diagnosis of the metabolic subtype of depression and to determine beneficial treatment strategies. By replicating the relationship between HOMA-IR and IMD symptoms, our study provides valuable insights into the early interaction between IR and IMD. This finding supports the use of HOMA-IR as a warning sign for IMD. Given the bidirectional link between IR and depression, our results suggest that individuals could benefit from a combined assessment of HOMA-IR and IMD symptoms.

Previous studies linked atypical depression to an unhealthy lifestyle and weight gain, potentially driven by hyperphagia^41^. In a community-based cohort, people with a remitted atypical-melancholic MDD subtype tended to be light movers, suggesting that they might particularly benefit from increased PA^43^. In contrast, we did not show an effect of PA on IMD symptoms. The divergence between our findings and those of previous studies demonstrating a beneficial effect of physical activity (PA) on depression^2,42,43^ can likely be attributed to differences in sample characteristics. While these prior studies primarily examined individuals with either an active or remitted episode of MDD, our sample comprised participants exhibiting symptoms of IMD without meeting the diagnostic criteria for a depressive episode. Further, alternative activity measures might better explain the associations between IR-related disorders and IMD symptoms^42^. Although our study did not establish a direct PA-IMD relationship, PA remains crucial for overall health benefits and diabetes prevention.

With respect to diet, we replicated the association between EDIP and CRP^54^, confirming EDIP’s ability to reflect dietary inflammatory potential. However, our study did not replicate prior findings linking a proinflammatory diet to general depression. This might stem from methodological differences. We are the first to explore a proinflammatory diet in the context of IMD rather than general depression or somatic symptoms. Moreover, previous studies often examined a proinflammatory diet using the DII, focusing on nutrients, while the EDIP emphasizes dietary patterns. Although both indices predict inflammatory markers, they are only modestly correlated^47^.

Notably, when examining the moderating effects of a proinflammatory diet, we observed sex-specific effects. Sex-specific effects have been reported by several studies^15,16,44^. However, these studies found a positive correlation between a higher dietary inflammatory index (DII) and depressive symptoms, with the effects being stronger and more consistent in women than in men^15,16,44^. Our results showed that in men, a proinflammatory diet strengthened the link between IR and IMD symptoms, whereas no significant relationship was observed in women (see Figs. 2and S3). A possible explanation for the sex-specific effects in our study might be a dose–response relationship. Luo et al^55^. recently suggested a non-linear dose–response relationship between a proinflammatory diet and symptoms of depression, with mildly proinflammatory diets reducing depressive symptoms. In our sample, the variance observed in the proinflammatory diet was limited, although it was comparable to previously reported EDIP ranges^47,54^. In addition, women had lower average EDIP values than men did. This may suggest that men might respond to lower thresholds of dietary inflammation, whereas in women, a proinflammatory diet might have little or no effect. Future studies should explore the sex-specific dose–response relationships between EDIP and IMD symptoms to gain a more comprehensive understanding of these interactions.

Although the moderation effects were statistically significant, their clinical magnitude should be interpreted with caution. In our sample, the IMD-score ranged from 0 to 5 (out of a possible 12), indicating generally mild symptom expression. A one-unit increase in the IMD-score reflects either the emergence of an additional immunometabolic symptom or an increase in the severity of an existing one. Thus, while the observed moderation effect (*β* = 0.72) suggests that diet-related inflammation may meaningfully amplify the burden of immunometabolic depressive symptoms among men with higher insulin resistance, the overall symptom level in this subclinical sample remained low. Future studies in clinical populations are needed to determine whether such effects correspond to clinically significant changes in depressive symptomatology.

Given the limited understanding of the IR-IMD relationship and the role of lifestyle, longitudinal studies with objective PA measures and reproducible dietary indices are needed. Although our results do not demonstrate a direct association between a proinflammatory diet and IMD symptoms, the sex-specific interaction of EDIP and HOMA-IR on symptoms of IMD suggests that further research is needed. Additionally, the observed association between IR and IMD needs to be replicated in a clinical sample to explore whether IR can help identify the IMD subtype. If such a link exists, lifestyle interventions may hold promise for reducing inflammation and increasing insulin sensitivity in individuals with depression characterized by IMD symptoms. This could serve as a valuable complement to anti-inflammatory and antidepressant medication and an important step toward precision medicine.

Our study has several strengths including its objectively recorded PA, detailed dietary assessment and a comprehensive analysis of the relationships between lifestyle factors, IR and IMD symptoms. However, several limitations must be taken into account. The small sample size and uneven sex distribution limit the generalizability of the findings and prohibit further subgroup analysis. In addition, the modest sample size may have limited the statistical power to detect small effects, particularly in sex-stratified analyses, and future studies with larger samples are warranted to confirm these findings. The cross-sectional design prevents temporal assessments and causal inference. Wrist-worn devices to record PA may influence usual behavior, warranting further exploration of measurement reactivity. Further, we had to exclude several individuals due to non-compliance with the sensor wear-time or technical difficulties. Moreover, validating the modified IMD-score used in this study is crucial to ensure reliability in future studies.

## Conclusion

Our results revealed a relationship between IR and IMD symptoms in an otherwise healthy population, suggesting that these conditions affect each other. Given the bidirectional link between IR and depression, it may be crucial to (1) monitor IMD symptoms in individuals at risk for IR-related disorders, and (2) use HOMA-IR as a biomarker to identify the IMD subtype. This could enable early detection and tailored treatment for these comorbid conditions. Our findings of sex differences in the modifying effects of diet on the relationship between IR and IMD suggest that men might benefit more from anti-inflammatory dietary interventions. Longitudinal studies and studies including a clinical sample are needed to better understand IR, lifestyle and IMD interactions and to identify effective treatments.

## Subjects and methods

This cross-sectional analysis used data from the *m*PRIME study, an observational study of the H2020 project PRIME (grant No. 847879) including a subclinical sample. Parts of the data of this study have been used for different research questions and details on the study methodology have been published elsewhere^51^. At baseline, sociodemographic data, depressive symptoms, blood samples, body weight, waist circumference and a 24-h-dietary recall were collected. During the ambulatory assessment, individuals wore an accelerometer for 7 days to record PA and completed three 24-h-dietary records on three consecutive days (from Thursday to Saturday or Sunday to Tuesday) to capture their habitual diet.

The study protocol and procedures were approved by the local ethics committee (reference number: 20–767) and all methods were performed in accordance with the Declaration of Helsinki^56^. The study was registered with the German Clinical Trials Register (DRKS00022774, Registration date: 2021-03-08). Informed consent was obtained from all individuals enrolled in the study.

### Study population

Briefly, in total 124 individuals (57 individuals with IR and 67 controls) were enrolled between March 2021 and March 2023, with the last participant completing follow-up in April 2024. The inclusion criteria were as follows: all participants were required to be 18 years of age or older and provide written consent for participation. Exclusion criteria included the use of antidiabetic medications or insulin; a diagnosis of type 1 diabetes mellitus and gestational diabetes; corticosteroid use; various psychiatric diagnoses including bipolar I disorder, schizophrenia, organically caused mental disorders, and substance dependence (other than nicotine and cannabis dependence); severe neurological disorders; pregnancy and breastfeeding; non-correctable visual impairments; participation in medication-related studies within the last 6 months; weight-reducing medications or a diet within the last 3 months; and language barriers. Six individuals dropped out of the study because of the substantial time burden and one individual was excluded because of existing unmedicated type 2 diabetes without meeting the criteria for IR. After plausibility and quality check, 30 participants with incomplete datasets (e.g. missing dietary, physical activity, or questionnaire data) were excluded from the analysis (described in the sections below). As a result, 94 individuals were included in the analyses. For this sample, missing data within variables were handled using pairwise omission, allowing all available information to be used in each analysis without excluding entire cases when data were partially missing.

### Blood samples

Blood samples were collected at baseline before the testing procedures by venipuncture after an overnight fasting period of at least 8 h. Details of the serum analyses (HOMA-IR and CRP) have been published elsewhere^51^.

#### Insulin resistance

Blood samples were sent to the hospital’s central laboratory to determine fasting insulin and fasting glucose concentrations. The HOMA-IR was calculated via the following formula: fasting insulin [mU/l] × fasting glucose [mg/dl])/405)^57^. HOMA-IR was transformed with the natural logarithm before analyses due to a right-skewed distribution.

#### Inflammation

CRP levels were measured to assess chronic inflammation. In addition, IL-6 was measured as part of the Olink Target 48 Cytokine Panel (Olink Proteomics, Uppsala, Sweden, the full panel will be reported elsewhere). Plasma was kept cool after blood sampling, and preprocessing occurred within 30 min. The OLINK 48 panel uses the Proximity Extension Assay (PEA) technology which has very high specificity and is highly reproducible. No levels were below the detection limits due to the high sensitivity of PEA (LOD:0.030 pg/mL).

Data for IL-6 were only available for a subset of individuals (*n* = 83). Due to a right-skewed distribution, both biomarkers were also transformed to the natural logarithm prior to analysis.

### Empirical dietary inflammatory pattern

Individuals reported food intake at baseline and during ambulatory assessment by completing one 24-h dietary recall and three 24-h dietary records, using the German version of myfood24^58^. Habitual food intake was assessed by averaging the dietary records/recalls. The EDIP was used to assess the inflammatory potential of the diet^47,54^. The EDIP is a continuous score based on servings of nine proinflammatory and nine anti-inflammatory food groups, with positive weights for proinflammatory and negative weights for anti-inflammatory groups. Total scores can be negative (indicating an anti-inflammatory diet), neutral or positive (indicating a proinflammatory diet).

Myfood24 includes two food composition databases for Germany – the German Food Code and Nutrient Database (BLS) and LEBTAB^58^. Both databases were used to extract information on food group classification and the amount consumed in grams. Foods recorded were assigned to one of the 18 EDIP food groups, while mixed dishes of the BLS were disaggregated as described. The consumed amounts (g) of each food were converted into serving sizes on the basis of the usual intake specified in the BLS, to apply the EDIP scoring system.

The dietary records of 124 individuals were available and were quality checked. Dietary records with < 600 and > 6000 kcal/day were excluded (*n* = 8 food protocols)^59^. Data from individuals were included in the analysis only if at least three food protocols passed the plausibility check. On this basis, seven individuals were excluded. On average, 3.93 ± 0.25 days of accuarte dietary protocols were available for the included individuals. EDIP scores outside the range (z-scores ≥ 3) were excluded from the analysis (*n* = 2), as they fell far outside previously reported EDIP ranges^47,54^.

### Physical activity

Details on the recording and operationalization of PA in the *m*PRIME study were published by Bruckner et al.^51^. In short, individuals wore an accelerometer (move3 or move4, movisens GmbH, Karlsruhe, Germany) on their non-dominant wrist for seven days and nights. Non-wear and sleep times were excluded from the calculation. PA data were available from 112 individuals. Individuals were included in the analyses only if they wore the sensor for at least 10 h on 5 days. Based on this, eleven individuals were excluded. Metabolic equivalents per minute were calculated via the software DataAnalyzer (movisens GmbH, Karlsruhe, Germany; version 1.13.7) and categorized as minutes spent with moderate (metabolic equivalent ≥ 3 to < 6) or vigorous (metabolic equivalent ≥ 6) PA. This was then summarized into one measure of PA by calculating the average minutes per day of moderate-to-vigorous PA (MVPA) for each individual. The average sensor wear time of the included individuals was 6.74 ± 0.6 days.

### Symptoms of immunometabolic depression

Depressive symptoms were assessed at baseline using the BDI-II^60^. The BDI-II is a 21-item questionnaire, with each item scored from 0 to 3. Cronbach’s alpha for the total score was 0.87, indicating high internal consistency in this sample. To examine IMD symptoms, we calculated an IMD-score by summing four items of the BDI-II, which have been previously found to be associated with immunometabolic changes and IR^12,24,29,32^. The four items are as follows: 15 = loss of energy, 16 = changes in sleep patterns, 18 = changes in appetite, 20 = tiredness. Changes in sleep patterns and appetite can occur in both directions (decrease/increase). Changes in sleep and appetite were considered only if they increased, indicating hypersomnia and hyperphagia. The IMD-score ranges from 0–12. The internal consistency for the IMD-score, as measured by Cronbach’s alpha, was 0.55, indicating a low level of internal consistency. Three individuals had incomplete questionnaires and were therefore excluded from the analysis.

### Statistical analysis

Descriptive information is presented as the means +/− SDs, and as medians [Interquartile ranges] or *n* (%). Mann–Whitney U tests and Pearson Chi-squared tests were performed to test for differences in sociodemographic characteristics between women and men. Spearman correlations were calculated to test the correlation between lifestyle factors and inflammatory biomarkers (CRP and IL-6), to assess the inflammatory potential of the lifestyle factors. Furthermore, Spearman correlations between inflammatory biomarkers (CRP and IL-6) and IMD were calculated, to assess whether the IMD-score was indeed associated with inflammatory changes. Missing data were handled using pairwise omission.

Three multiple linear regressions with the IMD-score as the dependent variable were performed to analyze the influence of IR and lifestyle on immunometabolic symptoms of depression. The first model included HOMA-IR, EDIP and MVPA as predictors. The second model included an interaction between HOMA-IR and EDIP and the third model included an interaction between HOMA-IR and MVPA to test for moderating effects of these lifestyle factors. Given the evidence indicating sex differences in dietary behaviour and the prevalence of IMD, as well as the emergence of sex as a predictor of IMD symptoms in the multiple linear regression analysis (see results section) we conducted explorative analyses stratified by participant sex. Models applied to the total sample were adjusted for age, sex (0 = male, 1 = female), smoking status (0 = non-smoking, 1 = smoking), BMI, hormonal contraceptive use (0 = no intake, 1 = intake) and menopause (0 = no, 1 = yes). Models applied only to men were adjusted for all covariates except for sex, hormonal contraceptive use and menopause. We tested the assumptions of the multiple linear regression models. The variance inflation factors (VIFs) were below 10, suggesting that there was no concern of multicollinearity. The Breusch-Pagan and Durbin-Watson tests supported the assumption of homoscedasticity and the absence of autocorrelation. However, the Shapiro–Wilk tests revealed that the assumption of normally distributed residuals was not met in each model, particularly when modeling was based on the total sample. To address these deviations, robust standard errors were calculated for each model using the “HC4” correction, which can be applied when the assumptions of homoscedasticity and normal distribution are violated^61^. A *p* value of < 0.05 was used to identify statistical significance. A post hoc power analysis indicated that the available sample size (N = 94 for the final analyses) provided approximately 80% statistical power to detect moderate effect sizes (f^2^ ≈ 0.15) in multiple linear regressions with up to nine predictors at an alpha level of 0.05. Although this sample size was sufficient for detecting medium effects, smaller effects may have gone undetected.

Given the exploratory nature of the study and the limited number of predefined hypotheses, no formal correction for multiple comparisons was applied. Instead, interpretation focused on effect sizes, consistency across models, and biological plausibility. We acknowledge that unadjusted *p*-values should therefore be interpreted with caution.

Data pre-processing and analyses were performed using SPSS (IBM, version 27.0.0.0), R (version 4.3.1; 2023-06-16 ucrt) and RStudio (version 2023.06.1.524).

## Supplementary Information

Supplementary Information.

## Abbreviations

BDI-II: Beck Depression Inventory-II
BMI: Body mass index
CRP: C-reactive protein
DII: Dietary inflammatory index
EDIP: Empirical dietary inflammatory pattern
HOMA-IR: Homeostasis Model Assessment of Insulin Resistance
IMD: Immunometabolic depression
IR: Insulin resistance
IRS: Insulin receptor substrate
MDD: Major depressive disorder
MVPA: Moderate-to-vigorous physical activity
PA: Physical activity

## Declarations

### Data availability

The datasets generated during the current study are available in the Zenodo repository, 10.5281/zenodo.11235373.
