# Supplementary material for: The moderating role of diet and physical activity in insulin resistance and immunometabolic depression
Source: Sci Rep. 2025 Dec 23;15:44417. doi: 10.1038/s41598-025-32454-4 (PMC12738732; doi:10.1038/s41598-025-32454-4)
Supplement: Supplementary file 1 — Supplementary Information. [file 41598_2025_32454_MOESM1_ESM.docx]

**Appendix**

**Table S1 – Spearman Correlations for Lifestyle Factors, Inflammatory markers and IMD-score**

| **Variables** | ***n*** | **EDIP** | **MPVA** | **CRP** | **IL-6** | **IMD-score** |
| --- | --- | --- | --- | --- | --- | --- |
| EDIP | 94 | 1 |  |  |  |  |
| MPVA | 94 | –0.11 | 1 |  |  |  |
| CRP^a^ | 94 | 0.279** | –0.435** | 1 |  |  |
| IL-6^a^ | 83 | 0.184* | –0.387** | 0.593** | 1 |  |
| IMD-score | 94 | 0.124 | –0.204* | 0.295** | 0.19 | 1 |

CRP, C-reactive protein; EDIP, Empirical dietary inflammatory pattern; IMD-score, immunometabolic depression score; IL-6, Interleucin 6; MPVA, moderate-to-vigorous-physical-activity.

^a^log transformed due to right skewed distribution.

**P* < 0.05. ***P* < 0.01


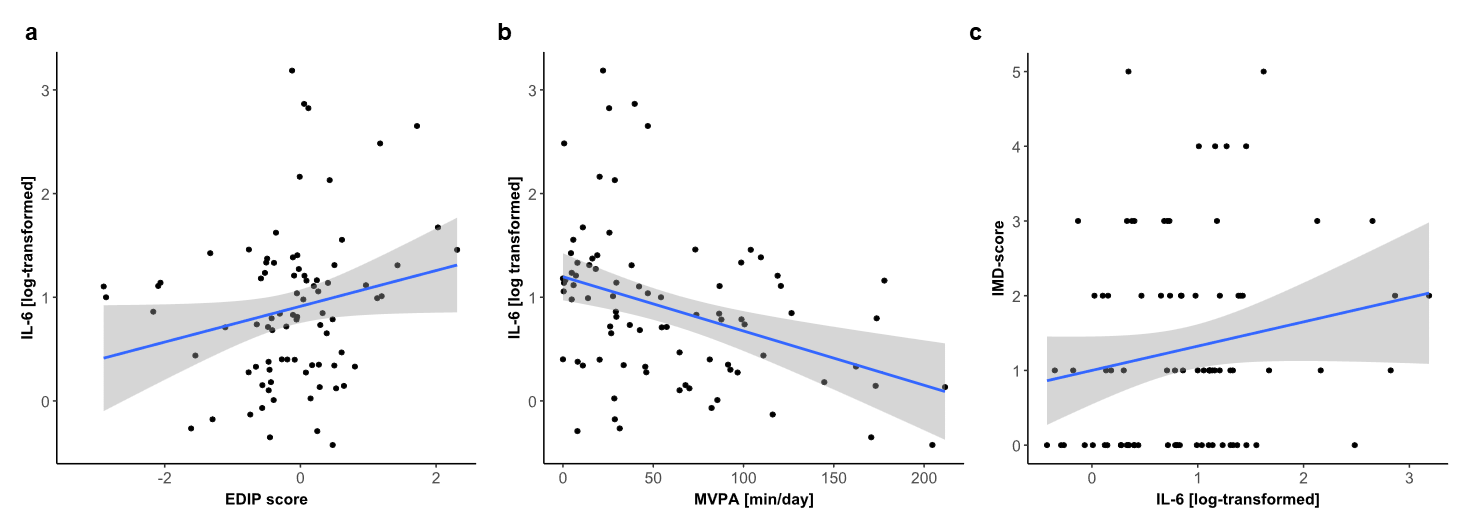


**Figure S1 - Relationships between IL-6 levels, lifestyle factors and IMD-score**

Association between: (a) EDIP (Empirical Dietary Inflammatory Pattern) and log-transformed IL-6 levels, (b) MVPA (Moderate to Vigorous Physical Activity) and. log-transformed IL-6 levels, (c) log-transformed IL-6 levels and IMD-score (immunometabolic depression score). All plots include a linear regression line with 95% confidence interval.


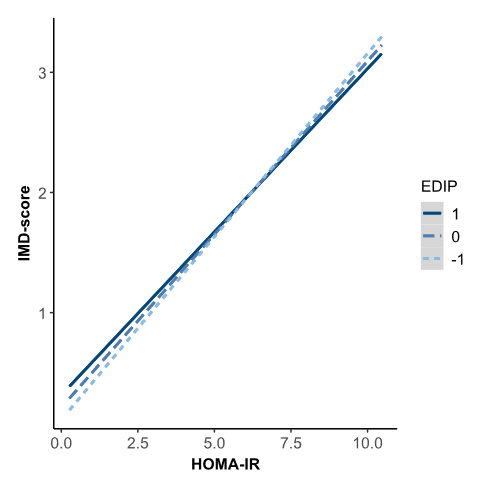


**Figure S2 – Interaction Plot between HOMA-IR and EDIP in the total population**

The x-axis represents HOMA-IR, while the y-axis shows the IMD-score (immunometabolic depression score). The interaction is shown for different EDIP (Empirical Dietary Inflammatory Pattern) scores defined as -1 (low), 0 (neutral) and +1 (high). The graph illustrates how variations in EDIP influence the relationship between HOMA-IR and IMD-score with separate regression lines for each score of EDIP.


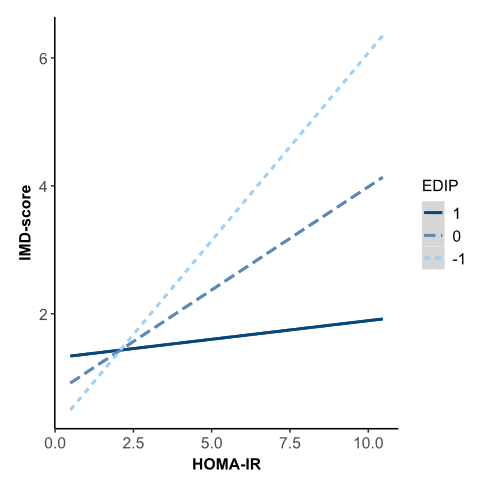


**Figure S3 – Interaction Plot between HOMA-IR and EDIP in women**

The x-axis represents HOMA-IR, while the y-axis shows the IMD-score (immunometabolic depression score). The interaction is shown for different EDIP (Empirical Dietary Inflammatory Pattern) scores defined as -1 (low), 0 (neutral) and +1 (high). The graph illustrates how variations in EDIP influence the relationship between HOMA-IR and IMD-score with separate regression lines for each score of EDIP.
